# Cavernous Sinus Thrombosis in Children

**DOI:** 10.15844/pedneurbriefs-30-1-3

**Published:** 2016-01

**Authors:** Rochelle Sweis, José Biller

**Affiliations:** 1 Department of Neurology, Loyola University Chicago, Stritch School of Medicine, Maywood, IL

**Keywords:** Carotid Arteries, Cavernous Sinus, Cerebrovascular Disorders, Infection, Magnetic Resonance Imaging

## Abstract

Investigators from the Children’s Hospital of Philadelphia analyzed the clinical and radiographic findings in 12 cases of cavernous sinus thrombosis (CST) seen between 2000 and 2013, and conducted a literature search and review of the pooled data.

Investigators from the Children’s Hospital of Philadelphia analyzed the clinical and radiographic findings in 12 cases of cavernous sinus thrombosis (CST) seen between 2000 and 2013, and conducted a literature search and review of the pooled data. Three of 12 (25%) cases experienced morbidity. Contrast enhanced MRI and contrast enhanced CT were 100% sensitive in detecting CST, while noncontrast MR venography and noncontrast CT were not sensitive (0% sensitive). The aggregate mortality rate in a total of 52 cases (12 plus an additional 40 cases from literature review) was 4 (8%) and morbidity rate was 10 of 40 (25%). Morbidity and mortality were low with early aggressive surgical, antimicrobial, and anticoagulation therapies, but outcome was not significantly changed. [1]

COMMENTARY. Pediatric cavernous sinus thrombosis (CST) is a rare and life-threatening complication of septic or aseptic etiologies, and is associated with low morbidity and mortality if aggressive therapies including antimicrobials, anticoagulation, and/or surgical treatment are implemented early on [1]. CST is associated with neurologic disability if not detected in a timely manner. Septic origin is often due to septic emboli or extension of a thrombophlebitis. The most common organism is Staphylococcus aureus (69%), followed by the Streptococccal species (17%), Pneumococcus (5%), gram negative species (5%), Bacteroides (2%), and Fusobacterium (2%). Valveless communications between the facial and ophthalmic veins and the cavernous sinus are responsible for infectious spread between the paranasal sinus and orbit to the cavernous sinus [2]. CST can also be a complication of otitis media and less often, pharyngitis or dental infection. The most common cause is acute sinusitis. Infections of the middle third of the face were responsible for most septic CST in the pre-antibiotic era though incidence has significantly decreased with the advent of antibiotic [3]. Aseptic CST is due to trauma or a pro-thrombotic etiology [3].

The most common presenting symptoms are due to the specific structures affected within the cavernous sinus. Compression of cranial nerves III, IV, and VI result in impaired extraocular movement with sixth nerve paresis being the most common.3 Compression of the ophthalmic and maxillary branches of cranial nerve V result in facial sensory deficits, periorbital sensory loss, and/or an impaired corneal reflex [3]. Unilateral periorbital edema, headaches, photophobia, chemosis, and proptosis are classic signs due to impaired venous drainage of the orbit [3, 4]. Papilledema, retinal hemorrhages, worsening visual acuity or blindness may also occur due to impaired venous drainage with resulting retinal congestion. Progression to bilateral orbital involvement due to an intercavernous communication, meningitis, subdural empyemas, and sepsis are common in CST [3]. Presence of Horner syndrome and sixth nerve paresis classically localize to the cavernous sinus. Systemic clinical features include pyrexia, tachycardia, hypotension, emesis, confusion, and even coma. Transient central hypopituitarism due to contiguous infectious spread leading to necrosis has also been reported with bilateral CST. Other complications of CST include infectious arteritis of the internal carotid artery, vasospasm, and infarcts, either embolic in origin or secondary to hypoperfusion [4]. Differentials include orbital cellulitis, intracranial infections, and superior ophthalmic vein thrombosis [5].
